# Long acting reversible contraception use and associated factors among married women of reproductive age in Nepal

**DOI:** 10.1371/journal.pone.0214590

**Published:** 2019-03-28

**Authors:** Rajan Bhandari, Khem Narayan Pokhrel, Nguyen Gabrielle, Archana Amatya

**Affiliations:** 1 Global Health Alliance Nepal (GHAN), Maharajgunj, Kathmandu, Nepal; 2 Institute for Reproductive Health, Georgetown University, Airport Gate Area, Shambhu Marga, Kathmandu, Nepal; 3 Save the Children, Washington DC, United States of America; 4 Department of Community Medicine and Public Health, Institute of Medicine, Tribhuvan University, Kathmandu, Nepal; Universidade Federal de Sao Paulo, BRAZIL

## Abstract

Modern contraceptives are highly effective and proven means of preventing unintended pregnancy and reducing maternal mortality. Social and economic characteristics are some of the key determinants of health and utilization family planning. However, studies examining the factors associated with utilization of long acting reversible contraception (LARC) are limited in Nepal. This study assessed the factors associated with utilization of LARC methods among married women of reproductive age in Nepal. Secondary data analysis was conducted using the 2016 Nepal Demographic and Health Survey (NDHS). A logistic regression model examined the association of socioeconomic, demographic, or fertility related characteristics with the use of LARCs among 9875 ever married women of reproductive age. The overall utilization rate of LARC in this study was 4.7%. Women in the age group of <25 years (AOR: 0.65, 95% CI: 0.45–0.92) and 25–35 years (AOR: 0.70, 95% CI: 0.56–0.89), having husbands with primary education (AOR:0.71; 95%CI: 0.64–0.84) and no education (AOR: 0.54; 95%CI: 0.38–0.73), belonging to Janajatis (AOR: 0.55; 95%CI: 0.42–0.71) and Newars (AOR: 0.29; 95%CI: 0.19–42), poor wealth quintile (AOR: 0.60; 95% CI: 0.45–0.86) had negative association with LARC use. On the other hand, women having their husband as a skilled worker (AOR: 1.49; 95%CI: 1.10–2), having two or less than two children (AOR: 1.46; 95% CI: 1.15–1.186), and having desire for children in future (AOR: 3.24; 95% CI: 2.29–4.57) had positive association with the use of LARC. In this study, younger women’s age, low or no husband’s education, from indigenous community such as Janajati and Newer, being in lowest wealth quintile negatively influenced the use of LARC. Conversely, women having her husband as skilled worker, parity less than two, and desire of having future children, positively influenced the use of LARC. The study highlights the need to reach women who were in the lower socioeconomic background to improve LARC use.

## Introduction

Family planning (FP) is fundamental to health of the women, their families, and community. Modern contraceptive methods are highly effective in preventing unintended pregnancies and reducing maternal and child mortality [1]. Long-acting reversible contraception (LARC) are methods of birth control that provide effective contraception for an extended period without requiring user action [2]. These methods can prevent as many as one in every three maternal deaths by allowing women to space births, avoid unintended pregnancies and abortions, and stop childbearing when they achieve their preferred family size [3]. Also, women who used LARC methods had lower return rates for rapid repeat abortions than women who used short acting methods [4–6].

The Implants and Intra-Uterine Copper Devices (IUCDs) are considered as a LARC. Women can prevent unwanted pregnancies at least for three years after using implants and for 12 years after using IUCD with the immediate return of fertility after removal of these devices [3]. These methods are considered to be more effective as they eliminate the risk of inconsistent and incorrect use that plague short-acting contraceptive methods as these require no user action after insertion [7]. Additionally, these methods are the most efficacious and cost-effective reversible forms of contraception, resulting in fewer pregnancies during the first year of use than any other reversible method [8].

The methods are highly effective, with pregnancy rates of less than one percent per year, and high rates of patient satisfaction and continuation [9]. With few exceptions, LARC has been proven safe for use by all women after menarche, including young women [10,11]. According to the Medical Eligibility Criteria (MEC) for contraceptive use outlined by World Health Organization (WHO) in 2015, adolescents (those ages 10 to 19) can have more sporadic patterns of intercourse, which may make non-daily methods, such as LARC, “more appropriate” for this group [12].

Nepal has prioritized family planning by enabling women and couples to attain their desired family size and have healthy and timely spacing of pregnancies. The country has set the goal to improve access to rights-based FP services and reduce unmet need for contraceptives. Nepal is working to improve the availability of a broader range of modern contraceptives and improved method mix, including LARC i.e. IUCD and implant at various levels of the health services, which is a recognized as a strong strategy for reducing unmet need for family planning and unintended pregnancy [13–15]. Despite these positive attributes, use of LARC methods among women of reproductive age (WRA) in Nepal is low at 4%, compared with the global utilization of 18.9% [16]. Among them, 3% of the women use implant and 1% use an IUCD in the country[17]. Although there has been a steady increase in overall contraceptive use from 29% in 1996 to 53% in 2016, the contraceptive prevalence rate for modern methods has remained stagnant from 2006 (44%) to 2016 (43%) [17,18]. This indicates that Nepal needs to strengthen its FP program to achieve the country’s commitments to global FP goals and to reach a modern contraceptive prevalence rate of 52% by 2020, the target set by Nepal Health Sector Strategy 2015–2020 (NHSS 2015–2020) [13].

Studies have indicated that various factors influence FP utilization, no matter the country context. Studies conducted globally have shown that the age, education, parity, future desire of children, access to media, and decision making power are the influencing factors for utilization of LARCs [3,19–25]. Moreover, a few studies in Nepal have shown that older women (35 and over), educated, living in urban areas, and working in the business or service sectors were more likely to use modern contraceptive methods [26]. Similarly, other studies conducted in Nepal has found that religion and exposed to media with family planning message were associated with the use of modern contraceptive methods [27,28]. However, most of the studies elsewhere have focused on examining factors that influence on the use of all modern contraception methods only. More specifically, evidence about the factors that influence the low uptake and use of LARC is limited in Nepal and elsewhere. Therefore, this study analyzed the nationwide survey data to examine the factors associated with utilization of long acting (Implant and IUCD) contraceptive methods among married women of reproductive age (15–49 years) in Nepal.

## Materials and methods

### Study design

The study was cross-sectional in design. The data were collected from a nationally representative sample collected for the Nepal Demographic and Health Survey (NDHS), 2016. We performed the secondary analysis of the survey data limiting to women who were currently using modern contraception for child limiting with specific focus on LARC methods.

### Sampling technique

The DHS survey used two-stage stratified cluster sampling in rural areas and three-stage stratified cluster sampling technique in urban areas, which was based on the updated version of the frame from the 2011 National Population and Housing Census (NPHC), conducted by the Central Bureau of Statistics (CBS). In rural areas, wards were selected as primary sampling units, and households were selected from the sample primary sampling units (PSUs). In urban areas, wards were selected as PSU, one enumeration area (EA) was selected from each PSU, and then households were selected from the sample EAs.

Seven provinces were stratified into urban and rural areas, yielding 14 sampling strata. Samples of wards were selected independently in each stratum. Implicit stratification and proportional allocation were achieved at each of the lower administrative levels by sorting the sampling frame within each sampling stratum before sample selection, according to administrative units at different levels, and by using a probability proportional to size selection during the first stage of sampling. Please see the NDHS 2016 for detail sampling process and technique [17] (URL: https://www.dhsprogram.com/Publications).

### Study participants and sample size

All married women aged 15–49 who were either permanent residents of the selected households or visitors who stayed in the households the night before the survey were eligible to be interviewed by DHS. In the interviewed households, 13,089 women aged 15–49 were identified for individual interview; interviews were completed with 12,862 women, yielding a response rate of 98%[17]. For this specific study, the final sample size was 9875 women who had currently married in the five year preceding the survey. The dataset of NDHS 2016 were available upon request (URL:https://www.dhsprogram.com/data/dataset/Nepal_Standard-DHS_2016.cfm?).

### Data collection tools and technique

Data were collected through face-to-face interview with eligible women using structured woman’s questionnaires. The questionnaires were developed and finalized in English according to the standard MEASURE DHS program guidelines [29] and they were translated into Nepali and other local language. Pretesting of questionnaire was performed with the help of trained and experienced enumerators.

### Ethical considerations

The standard MEASURE DHS guidelines about the ethical consideration were followed to collect information for NDHS 2016. The survey protocol was reviewed and approved by the Nepal Health Research Council (NHRC) and the ICF Institutional Review Board. The survey data were made available with a request to ICF.

### Variables and operational definition

#### Outcome variable

Outcome variable for this study is a binary response indicating whether a woman used a LARC method (IUCD and Implant). The LARC use was recoded as a dichotomous variable with value 1 for “LARC use” and 2 for “not use” for logistic regression analysis.

#### Exposure variables

For this study, a number of exposure variables were selected based on the theoretical and empirical significance shown in different studies and selected variables and grouped into composite measures as described below:

Age group: categorized with value of 1 for>35 years, 2 for 25–35 years and 3 for <25 years[17].
Number of live children: described in two categories with value of 1 for ≥ 3 Children and 2 for ≤ 2 Children[17].
Education of women: created by recoding the education in three categories with value of 3 for “no education”, 2 for “incomplete secondary and complete primary education” and 1 for “complete secondary and higher education”[17].
Education of husband: described in four categories with value 4 for “no education”, 3 for “primary education”, 2 for “secondary education” and 1 for “higher education”[17].
Religion: described in two categories with value 1 for “Hindu” and 2 for “other religion” comprising Buddhist, Muslim, Kirat, Cristian, and other[17].
Ethnicity and casts: created by recoding 11 castes into five categories with value of 1 for “Brahmin and Chhetri” which included Brahmin and Chhetri of Hill and Terai, 2 for “Janajati” comprising Janajati of Hill and Terai, 3 for “Newari”, 4 for “Dalit” including Dalit if Hill and Terai, and 5 for “other religions” which included Terai casts, Muslim and other[17].
Occupation of women: described in three categories with value 1 for “not working”, 2 for “agriculture” and 3 for “other occupations” which included professional/technical/managerial, clerical, sale/service, household and domestic, service, skilled and unskilled manual and others[17].
Occupation of husband: described in four categories with value 1 for “agriculture”, 2 for “Professional/Technical/ Managerial and Clerical”, 3 for “Skilled manual” and 4 for “Other occupation” comprising unskilled manual, sales/services, not working and others[17].
Wealth index: categorized five household wealth quintiles into 3 categories with value 1 for “wealthiest” including poor and poorest quintile, 2 for “middle” and 3 for “least wealthy” including rich and richest quintile as there was no difference in two categories [17].
Media access with FP message: is a composite variable created by combining whether the respondent reads family planning messages in newspaper, magazine, listen to radio and watch in TV with a value of 3 for “no access” if a woman has no access to FP message through either of the three media, 2 for “medium access” if a woman has access of FM message through either of three media and 1 for “high access” if a woman has access of FP message through more than two media at least once a week[17].
Future desire of children: created by recoding variable into two categories with a value of 2 for “have another child” and 1 for “no more children”[9,17,22]
*Knowledge about fertile days*: created by recoding variable into two categories with a value 2 for “yes” and 1 for “no”[17]

### Data analysis

Bivariate analysis was performed to examine the strength of association between LARC use and the selected individual characteristics in terms of Crude Odds Ratio (COR). Multivariate analysis was done using multiple logistic regression model. In the model, the LARC use was an outcome variable after adjusting for participant’s age, religion, ethnicity, number of live children, wealth tercile, occupation of husband, education of husband, occupation of women, education of women, and future desire of children as independent variables. The associations were reported in terms of Adjusted Odds Ratio (AOR) with their p-values and 95% confidence interval (CI) after controlling for potential confounders. Analysis was performed using IBM SPSS version 22.

## Results

**Fig 1:**
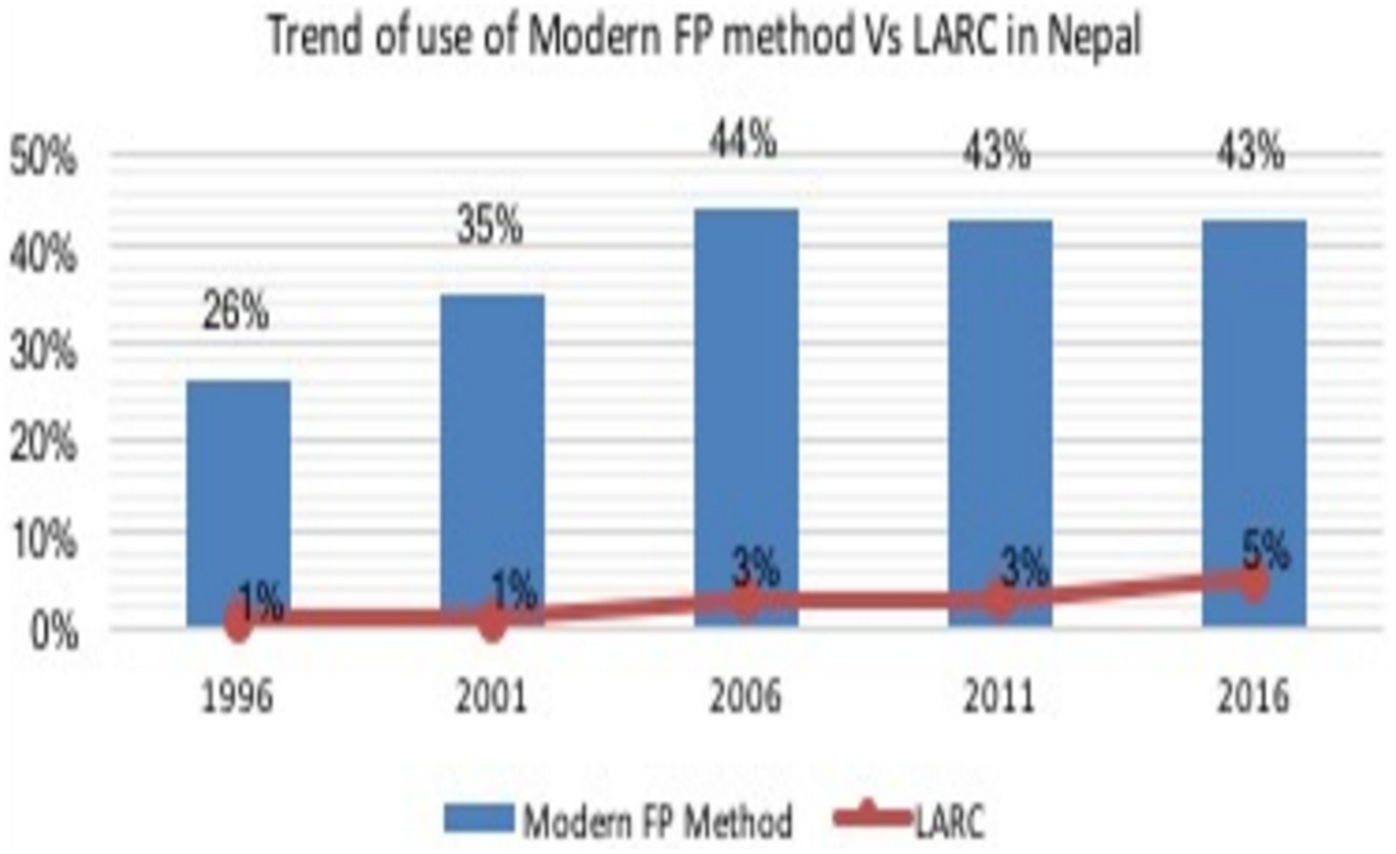
Trend of use of modern FP methods Vs LARC in Nepal.

Out of 9875 married women, 42.8% (n = 4225) used any modern method FP, which has remained almost stagnant since 2006. Similarly, the use of LARC in 2016 was 4.7%(n = 468), in which the use of IUCD was 1.4% (n = 139) and implant was 3.4% (n = 329). Use of LARC has slightly been increased to 5% in 2016 in comparison with 3% in 2011 (Fig 1).

However, the use of LARC is varied according to different socio-demographic and economic characteristics of women (Table 1). The proportion of LARC use within the age of participants was almost similar among women with the age groups 25–35 years (5.3%) and >35 years (5.2%) which higher compared to women with age group 20–24 years (3.4%) and adolescent women (age below 20 years) 2.4%. Similarly, use of LARC was higher (6.3%) among women who had three or more live children compared with the women who had less than three children (3.7%). About educational status, the proportion of LARC use was higher for those women who had no education (5.6%) compared to those women who had education level secondary and above (3.9%). Likewise, the use of LARC was also higher among women whose husband had primary education (6.4%) and no education (5.6%) compared to those who had higher education (4%). With respect to the occupation, the proportion of LARC use was higher among women who worked in agriculture (5.7%) compared to not working women. Similarly, LARC use was 4.1% among women whose husband had worked as a professional/technical/managerial and clerical (4.2%) followed by skilled manual. The proportion of LARC use was higher among women who belonged to Hindu religion (4.9%). (Table 1).

**Table 1:** Socio-demographic and economic characteristics and use of LARC.

Characteristics	LARC use		Total women (n) weighted	COR (95%CI)	p-value
	Number	Percent			
**Age**					
>35 years	186	5.3	3477	1	
25–35 years	208	5.2	4009	1.03 (0.84–1.27)	.757
<25 years	74	3.1	2389	1.78 (1.35–2.33)	< .001
Total	468	4.7	9875		
**No. of children (live)**					
≥ 3 Children	241	6.3	3805	1	
≤ 2 Children	227	3.7	6070	0.75 (0.56–1.00)	.057
Total	468	4.7	9875		
**Education of women**					
Complete Secondary and above	73	3.9	1861	1	
Incomplete secondary & Primary	171	4.2	4030	0.92 (0.69–1.21)	.550
No education	224	5.6	3984	0.67 (0.52–0.90)	.006
Total	468	4.7	9875		
**Education of Husband**					
Higher	80	4.5	1783	1	
Secondary	158	3.6	4337	1.24 (0.94–1.63)	.130
Primary	137	6.4	2157	0.68 (0.52–0.91)	.010
No Education	94	5.9	1598	0.8 (0.6–1.0)	.069
Total	469	4.7	9875		
**Religion**					
Hindu	419	4.9	8552	1	
Other*	49	3.7	1322	1.43(1.06–1.94)*	.019
Total	468	4.7	9874		
**Ethnicity**					
Brahmin and Chhetri	122	4	3073	1	.005
Janajati	196	6.6	2957	0.59(0.48–0.75)	< .001
Newari	45	10	452	0.41(0.29–0.58)***	< .001
Dalit	53	4.2	1265	0.91(0.66–1.27)	.581
Others**	53	2.5	2128	1.48(1.07–2.04)*	.019
Total	469	4.7	9875		
**Occupation of women**					
Not Working	106	3.4	3141	1	
Agriculture	276	5.7	4802	0.53(0.42–0.67)***	< .001
Others***	86	4.5	1931	0.74(0.56–0.99)*	.042
Total	468	4.7	9874		
**Occupation of Husband**					
Agriculture	153	7.1	2161	1	
Professional/Technical/Managerial and Clerical	165	4.2	3970	1.75(1.39–2.19)***	< .001
Skilled Manual	74	4.1	1787	1.76(1.32–2.33)***	< .001
Others****	76	3.9	1956	4.79(3.63–6.34)***	< .001
Total	468	4.7	9874		
**Wealth index**					
Wealthiest	144	3.5	4154	1	
Middle	75	3.6	2088	0.97(0.73–1.28)	0.813
Least wealthy	249	6.9	3632	0.49(0.40–0.60)	< .001
Total	468	4.7	9874		
**Media access with FP message**					
High access	104	4.7	2230	1	
Medium access	135	5.1	2637	0.91(0.70–1.18)	0.477
Low access	229	4.6	5007	1.02 (0.80–1.30)	0.834
Total	468	4.7	9874		

Other* = Buddhist, Muslim, Kirat, Christian and other; Other** = Other Terai caste, Muslim and other; Other*** = Did not work, Unskilled manual, Household, service, skilled and unskilled manual and other; Other**** = Did not work, Unskilled manual, Household and other.

### Findings from bivariate analysis

#### Sociodemographic and economic characteristics with LARC use

Women’s demographic and socio-economic characteristics such as age of women, education of women, education of husband, religion, ethnicity, occupation of women, occupation of husband, household wealth quintile including fertility related characteristics such as future desire of children were significantly associated with the use of LARC as a method of contraception. Compared with the women of age more than 35 years, the women with age group <25 years had higher odds of using LARC (COR: 1.78; 95% CI: 1.35–2.33). Similarly, women with no education were less likely to use LARC by 33% compared to women who had secondary education and above (COR: 0.67; 95% CI: 0.52–0.90; p<0.006). Women with their occupation as agriculture (COR: 0.53; 95%CI: 0.42–0.67) and other occupations (COR:0.74; 95%CI: 0.56–0.99) had lower use LARC by 47% and 26%, respectively.

Regarding husband characteristics, women having their husband with primary education were less likely to use LARC by 23% compared to those having husband with higher education (COR: 0.68; 95% CI: 0.52–0.91). Whereas, the odds of using LARC among women having their husband with occupation as skilled manual (COR: 1.76; 95%CI: 1.32–2.33), profession/technical/managerial (COR: 1.75; 95% CI: 1.39–2.19) and other occupations (COR: 4.79; 95%CI: 3.63–6.34) was higher in comparison to the women having their husband working as an agriculture. Women from other religions such as Buddhist, Muslim, Kirat, Christian were 1.4 times more likely to use LARC compared to those from Hindu religion (95%CI: 1.06–1.94). Women from household with least wealthy wealth quintile were less likely to use LARC as a contraceptive method by 51% compared to those from households with wealthiest quintile (COR: 0.49; 95%CI: 0.40–0.60) (Table 1).

#### The association of fertility characteristics and LARC use (unadjusted analyses)

The odds of LARC use was 3.4 times higher among women who wish to have a child in the future compared with the women who wish to have no children (95% CI: 2.52–4.64). While there was no statistically significant difference in LARC use among women who had knowledge about fertile days and those who did not have (Table 2).

**Table 2:** Fertility related characteristics and use of LARC.

Characteristics	LARC Use		Total Women (n) weighted	COR (95%CI)	p-value
	Number	Percent			
**Future desire of Children**					
No more	421	5.8	7213	1	
Have Another in future	47	1.8	2662	3.42 (2.52–4.64)	<0.001
Total	468	4.7	9875		
**Knowledge about fertility**					
No	343	4.8	7210	1	
Yes	125	4.7	2664	1.01 (0.82–1.25)	0.909
Total	468	4.7	9874		

#### Findings from Multivariate analysis

Unadjusted and adjusted odds ratio for LARC use are outlined in Table 3. After controlling sociodemographic (age, religion, ethnicity, number of live children, wealth, occupation of husband, education of husband, occupation of women, education of women) and fertility related characteristics (future desire of children), women who were in the age group of <25 years (AOR: 0.65, 95% CI: 0.45–0.92) and 25–35 years (AOR: 0.70, 95% CI: 0.56–0.89) were less likely to use LARC compared to women more than 35 years of age (Table 3).

**Table 3:** Effects of socio-demographic and economic and fertility related characteristics on use of LARC.

Characteristics	Number (%) of LARC use	Total Women (n) Weighted	COR (95%CI)	AOR (95%CI)
**Age of women**				
>35 years	186 (5.3)	3477	1	1
25–35 years	208 (5.2)	4009	1.03 (0.84–1.27)	0.70 (0.56–0.89)**
<25 years	74 (3.1)	2389	1.78 (1.35–2.33)***	0.65 (0.45–0.92)*
**No. of live children**				
≥ 3 Children	241 (6.3)	3805	1	1
≤ 2 Children	227 (3.7)	6070	0.75 (0.56–1.008)	1.46 (1.15–1.86)**
**Education of women**				
Complete Secondary and above	73 (3.9)	1861	1	1
Incomplete secondary & Primary	171 (4.2)	4030	0.92 (0.69–1.21)	1.18 (0.86–1.64)
No education	224 (5.6)	3984	0.69 (0.52–0.90)**	1.14 (0.78–1.66)
**Education of Husband**				
Higher	80 (4.5)	1783	1	1
Secondary	158 (3.6)	4337	1.24 (0.94–1.63)	1.50 (0.90–2.05)
Primary	137 (6.4)	2157	0.69 (0.52–0.91)**	0.71 (0.64–0.84)**
No Education	94 (5.9)	1598	0.75 (0.55–1.02)	0.54(0.38–0.73)*
**Religion**				
Hindu	419 (4.9)	8552	1	1
Other	49 (3.7)	1322	1.43(1.06–1.94)*	1.37(0.99–1.89)
**Ethnicity**				
Brahmin and Cheetri	122 (4)	3073	1	1
Janajati	196 (6.6)	2957	0.59(0.48–0.75)	0.55(0.42–0.71)***
Newari	45 (10)	452	0.41(0.29–0.58)***	0.29(0.19–42)***
Dalit	53 (4.2)	1265	0.91(0.66–1.27)	1.03(0.72–1.47)
Others	53 (2.5)	2128	1.48(1.07–2.04)*	1.37(0.95–1.96)
**Occupation of women**				
Not Working	106 (3.4)	3141	1	1
Agriculture	276 (5.7)	4802	0.53(0.42–0.67)***	0.92(0.71–1.18)
Other	86 (4.5)	1931	0.74(0.56–0.99)*	0.92(0.68–1.24)
**Occupation of Husband**				
Agriculture	153 (7.1)	2161	1	1
Professional/Technical/Managerial and Clerical	165 (4.2)	3970	1.75(1.39–2.19)***	1.29(0.99–1.69)
Skilled Manual	74 (4.1)	1787	1.76(1.32–2.33)***	1.49(1.10–2)**
Others	76 (3.9)	1956	4.79(3.63–6.34)***	1.54(1.14–2.06)**
**Wealth index**				
Wealthiest	144 (3.5)	4154	1	1
Middle	75 (3.6)	2088	0.97 (0.73–1.29)	0.94 (0.69–1.28)
Least wealthy	249 (6.9)	3632	0.49 (0.40–0.60)***	0.60 (0.45–0.86)***
**Future desire of Children**				
No more	421 5.8	7213	1	1
Have Another	47 1.8	2662	3.42 (2.53–4.64)***	3.24 (2.29–4.57)***

Statistically significant at

*p<0.05,

**p<0.01,

***p<0.001,

CI = Confidence Interval, COR = Crude Odds Ratio, AOR = Adjusted odds Ratio, 1 = Reference, Adjusted variables: Age, Religion, Ethnicity, Number of live children, Wealth, Occupation of husband, Education of husband, Occupation of women, Education of women, Future desire of children

Similarly, women who had two or less than two children were 1.5 times more likely to use LARC (95% CI: 1.15–1.186) compared to women who had three or more live children. Although the proportion of LARC use among women with no education was higher and shown to be associated in bivariate analysis, after controlling for sociodemographic and fertility related characteristics, the difference was not statistically significant (AOR: 1.14; 95% CI: 0.78–1.66). On the other hand, husband’s education level was associated with LARC use. Women with husbands with primary education and no education were less likely to use LARC by 29% and 64% respectively (AOR:0.71; 95%CI: 0.64–0.84) (AOR: 0.54; 95%CI: 0.38–0.73) compared to those women whose husband have higher level of education (Table 3).

Ethnicity was also associated with use of LARC. Women who belonged to Janajati (AOR: 0.55; 95%CI: 0.42–0.71) and Newari (AOR: 0.29; 95%CI: 0.19–42) ethnic groups were less likely to use LARC compared to Brahmin and Cheetri. Women whose husbands worked as a skilled manual laborer were 1.5 times (AOR: 95%CI: 1.10–2) more likely and those whose husbands worked as other occupation like unskilled manual, household work and who did not work, were 1.5 times (AOR: 95%CI: 1.14–2.06) more likely to use LARC compared to women having their husband working in agriculture. Women from households in the least wealthy wealth quintile were 40% less likely to use LARC compared to those belonged to the households with wealthiest wealth quintile (AOR: 0.60; 95% CI: 0.45–0.86). While looking at the fertility related characteristics, women who had a desire of another child in the future were more likely to use LARC compared to those who did not have (AOR: 3.24; 95% CI: 2.29–4.57) (Table 3).

## Discussion

In this study, sociodemographic characteristics such as age of women, ethnicity, number of live children, wealth quintile, occupation of husband, education of husband were the contributing factors of LARC use. Additionally, women who had future desires for children were more likely to use LARC compared to those who did not want children in the future.

This study found that younger women were less likely to use LARC in comparison to older women. This might be due to that women with younger age preferred to use short term or natural method of family planning. In Nepal, studies reported that younger women with fertility intention feel a fear of side effects or have misconceptions such as LARCs causing infertility and IUCD may move towards heart from uterus [30]. However, our results do not match with studies conducted in Ethiopia and Iran [31,32]. Other studies conducted in various countries found that age was not significantly associated with the use of LARC[3,8,23,24,33]. This study provides the evidence that, in Nepal, women’s age is one of the contributing factors of LARC use.

Regarding parity, women who had two or less children were more likely to use LARC compared to those women who had three or more live children. This finding is consistent with the studies conducted in Ethiopia and Iran [24,32,34]and contradicts with the other studies conducted in Ethiopia and Uganda [3,33]. Women and couples who believed that two or a smaller number of children would not be sufficient in their life, might have wanted to space the pregnancies and hence preferred using a LARC method. Our findings suggest that lower parity is also a factor that influences use of LARC in Nepal.

LARC use progressively decreased with wealth quintiles. The women who belonged to households in the lowest wealth quintile were less likely to use LARC compared to women who belonged to the highest wealth quintile. This result is consistent with the studies conducted in Ethiopia, Iran, and Nigeria respectively [22,32,35], as these studies found the positive association LARC use with higher wealth quintile. This could be due to limited access to information regarding LARC in women of lower wealth quintiles [17].

Desire of children in the future was positively associated with use of LARC. Women might have decided to use LARC with the understanding that LARC would be reversible nature and fertility would return immediately after stopping use of these methods [36]. This finding is consistent with the results of studies conducted in Ethiopia [3,22,34]. As in other countries, this is the additional evidence in Nepal as desire for future children positively influences the use of LARC.

Husband’s education also influenced the use of LARC. Women having husbands who had primary education and no formal education were less likely to use LARC compared to those women whose husband had higher level education. More educated men might have known the various methods of FP and their benefits including positive decision making on LARC use. Also educated husband of women do have decision making practices together[17]. Our results do not align with the studies conducted in other countries as these studies had no significant association between husband’s education and use of LARC [3,22,24]. This is the novel evidence in Nepal that husband’s education level is the influencing factor on use of LARC.

In this study, occupation of husband had positive influence on the LARC use. Women who had their husband as skilled worker had higher use of LARC compared to those in the agriculture profession. Skilled work might have contributed to the use of FP resources and information as they had exposure to the government health services. Also, service holders husband might have involved in decision making with their wife. Women who belonged to Janajati and Newar ethnic groups were less likely to use LARC compared to Brahmin and Chhetri. This result is likely related to the fact that most of the women from these ethnic minority groups may have less involved in FP decision making, and have less access to FP services.

Although, education of women was not significantly associated with the use of LARC, educated women had higher proportion of use. The result might have been affected by the lower sample size of educated women compared to non-educated women. However, studies conducted in Ethiopia, Iran, Nigeria [3,8,23–25,32] had similar results. Studies have suggested that greater gender equality may encourage women’s autonomy and may facilitate the uptake of contraception because of increased female participation in decision making. Autonomy of women influences contraceptive use in Asian population [37,38].

The study has major two limitations. First, the study only captured the information of DHS data. The context specific data may provide better picture of the LARC use as the country has diverse background in terms of social norms. However, the study can be generalizable as it represents the whole country. Second, this study only captured the service user’s characteristic and access to LARC services, availability of skilled service providers to administer LARC and service provider’s perspectives were not included. Similarly, some of the user perspectives like past experiences of LARC use and experiences of side effects of contraception use may also contribute to the use of LARC which is not included in this study due to the limitation of DHS data. However, this study tried to capture the women’s and husband’s characteristics, including fertility preferences, that may influence care seeking from the service centers. More studies are warranted, to explore the barriers of service use including the health system barrier and perspectives of service providers.

## Conclusions

LARC use was influenced by women’s and their husband’s sociodemographic characteristics and desire of future children. More specifically, younger age women, those with low parity, having uneducated husband and, being in the household with low wealth quintile were less likely to use LARC. It is also noteworthy that the desire of children in the future was positively associated with the LARC use. Thus, future interventions should be designed to reach women with younger age groups, lower socioeconomic backgrounds, and having husband with low or no education in order to improve the LARC use.
